# Practical Medicine

**Published:** 1876-08

**Authors:** 


### IV. Practical Medicine.

1. *Fork in the Stomach — Gastrotomy — Cure.* Labbe. (*L'Union Médic.*, April 27, 1876.)

A young man eighteen years old, desiring to imitate a juggler, swallowed a fork, whose points were held by his teeth. This he did with impunity several times, but finally, in consequence of a sudden movement induced by some pleasantry on the part of his companions, the part between his teeth slipped away and the foreign body lodged itself deeply in the pharynx. Neither he nor his affrighted companions could seize the fork with the fingers. Dr. Lepère managed to seize the prongs with a long pair of polypus forceps, but the patient pushed him violently away in consequence of the pain excited, when the fork buried itself still deeper in the œsophagus. The patient soon became free from pain and even jocose over his predicament.

In a fortnight, intense pain occurred with syncope, after the relief of which appeared a tumor of considerable size over the large extremity of the stomach. Each meal was succeeded by severe pain. A year passed, during which he had intervals of great pain and comparative comfort. Six months of this period were employed in pursuing his ordinary avocation.

At this time, by means of certain exact manœvres, he could make the prongs of the fork project between the epigastrium and the hypochondrium, so that it could be very distinctly recognized through the abdominal parietes—the act being more successful when the stomach was distended with aliment. His health and spirits were now profoundly affected.

Gosselin, Larrey and Lepère agreed, after consultation, upon surgical interference by the aid of caustics, but could not succeed in producing adhesions between the stomach and abdominal walls with either the paste of Canquoin or Vienna.

After numerous experiments and studies upon the cadaver, Gosselin, Larrey, Lepère, Coyne and Mene-Maurice assisted the reporter in performing gastrotomy, April 9.

After anæstheziation by chloroform, L. incised each layer separately, to the extent of 4 ctm., in a line extending parallel to the false ribs of the left side and 1 ctm. distant, and terminating in the imaginary line uniting the cartileges of the ninth ribs. Six successive applications of caustic had been previously made in this locality. The wound was kept open and the visceral and parietal layers of peritoneum were found ununited.

The anterior wall of the stomach was seized and drawn somewhat into the wound by a pair of forceps, then a loop of thread was passed through the fold and the latter brought into firm apposition with the lips of the abdominal wound. Eight points of suture were then made with strongly curved needles, through the stomach and abdominal walls — the point of each needle entering the stomach from without inward and escaping in an inverse direction. Thus the visceral and parietal layers of peritoneum were firmly apposed to the extent of 1 ctm. almost the entire periphery of the wound.

The stomach was then incised, the foreign body recognized (the prongs fastened in a mass of spongy tissue to the left of the wound in the greater curvature), and after exploration with a pair of long polypus forceps, the fork was seized and readily extracted.

Peritonitis was threatened, but a collodium "cuirass" upon the abdomen, with iced champaigne internally, was followed by such improvement that in five days solid aliment was ingested. The cure is now complete, save for the existence of a rapidly contracting gastric fistula, which scarcely admits the little finger.

2. *Râle of the Buccal Cavity.* GALVAGNI. (*Annali Unio. di Medic.*, Jan., 1876.)

Some phthisical patients are conscious of a râle in their own persons, which they refer to the base of the neck, toward the fourchette of the sternum, and of which they sometimes complain bitterly. This little râle is often perceived by persons who approach them and who are intimately associated with them.

Physicians can readily appreciate this sound by bringing the ear near the mouth of the patient. It is small, fine and dry, and is caused by the breaking of one or more small bubbles. It is sometimes audible during inspiration, more often it coincides with the two periods of respiration. Sometimes, though rarely, auscultation of the mouth reveals ronchus and whistling.

At first, one is tempted to localize these sounds in the upper part of the trachea, because of their clearness and often of their intensity; a more attentive examination, however, discloses the fact that they arise more deeply in the lungs. Indeed, if the trachea be examined by the ear with the patient's mouth closed, nothing is heard. The sound reappears as soon as the mouth is reopened. This shows that the sound is engendered deeper than the trachea, and that it is transmitted by the column of air. Beside, when auscultation is made comparatively of the trachea and different parts of the thorax, the sound is discovered in the latter having the same characters as that in the mouth; the bubbles are only a little finer, dryer and somewhat more high-pitched in the latter case. This is due to the influence exerted by the head cavity upon the sound which it transmits. It is for this reason also that the sound is more distinctly audible near the mouth. Not only is this sound reinforced by the cavity of the mouth and fauces, but also, if it be central in origin, it may be marked to thoracic auscultation by the interposition between it and the ear of pulmonary bubbles full of air.

G. has not only discovered this râle in phthisis, but also (rarely) in pneumonia, in four cases of capillary bronchitis, in one case of pleural exudation, and in cases of pulmonary vomicæ. He attributes to it a very small diagnostic value, but thinks it might be of service in cases of incipient pulmonary phthisis, when thoracic auscultation gives unsatisfactory results. Its greatest value would appear to be in veterinary practice, where thoracic auscultation is often surrounded with great difficulties.

3. *Treatment of Variola by Exclusion of Solar Light.* WATERS AND GADDESDEN. (*Abeille Médic.*, *Lyon Médic*, May 21, 1876.)

The two authors first published, in 1870 and 1871, their experiments in the treatment of the eruptive fevers by the exclusion of solar light—covering completely the shutters and windows of the patient's chamber with opaque material and lighting the apartment solely by a candle or oil lamp. They concluded that if the daylight were completely excluded from the chamber of the variolous patient the disease became incontestably less grave, the skin affection was arrested at the vesicular stage, pus was not formed, the interspaces between the variolous vesicles did not inflame, crusts did not form, the pain was diminished, the pruritus became insignificant and the odor diminished. *But, if during the period of primitive fever or eruption, the light was admitted to the chamber, even for a few instants, grave consequences followed, and the good effects previously produced were sometimes neutralized.*

Dr. Patin, of Orse, made trial of this treatment in 1870 and 1871, in the cases of seven patients attacked during an epidemic, with encouraging results, as will be seen from the following details:

I. Mme. E. L., non-vaccinated. Delivered on the evening of May 26, 1871. Attacked with confluent variola the next day. May 31, convalescence; fever and eruption arrested.

II. The daughter of this lady, fifteen days old, had confluent variola, June 8. Treatment by obscurity. On the fifth day there was great improvement; but on the 7th day the mother took her out of the darkened chamber to show her to a neighbor, contrary to orders. The variola recurred with intensity, and the child died in the night.

III. Mme. de V., aged fifty-seven years, vaccinated, confluent variola May 31; obscurity June 8; convalescence and no traces of variola.

IV. Miss E. B., aged twenty years. Sent home to her friends June 29th with commencing variola, (very violent fever, rachialgia, variolic pustules upon the upper lip.) Complete obscurity; defervescence in twenty-four hours. For nine days she took supper with the family, after night-fall. At the expiration of this period she was as "fresh looking as if nothing had occurred."

V. Miss L. G., aged eighteen years. Confluent variola five days after, July 12. Obscurity; convalescence in eight days, without any facial appearances of disease.

VI. Mr. M. L., June 29, confluent variola, (not recognized at the outset.) Repeated bleeding and purging, and finally general prostration with administration of the last sacrament. After a fortnight of obscurity he was cured without facial deformity, but there was suppuration upon lower extremities, resulting from prolonged use of sinapisms.

VII. M., non-vaccinated infant, one year old. Discrete variola after three days invasion. Obscurity; cure in eight days, (having taken no medicine,) without appearance of cicatrices.

These cases are too few, suggests the reviewer, for the purpose of demonstration. He inquires, also, whether it would not be well to experiment with other eruptive fevers, puerperal peritonitis, septicaemia, etc., as well as to investigate the history of these and similar disorders in the localities where the Arctic night occurs. The treatment does not preclude proper medication.

4. *Diagnosis of Fissures of the Anus in Children at the Breast.* MARBOUX. (*L'Union Médic.*, No. 61.)

Three affections—hæmorrhoids, polypus of the rectum, and fissure of the anus, are accompanied by pain and hæmorrhage, in connection with defecation. Exploration of the anus is indispensable in order to arrive at a diagnosis. This should be performed with great delicacy, so as not to increase the rent (when it exists), and especial care should be taken in completely separating the folds of the mucous membrane, for fissure is always found between two folds, and, when small and recent, is liable to be overlooked in a superficial examination.

Polypus of the rectum is ordinarily seen when the anal opening is examined at the moment of defecation, in the form of a small, red, mamelonnated enlargement, somewhat resembling a raspberry. External hæmorrhoids are readily seen, but then the folds of membrane should always be separated for inspection, as fissure may also be present. Internal hæmorrhoids may also be seen through the anal orifice in the efforts of defecation; and sometimes are with difficulty, and only after several examinations, dissociated from polypus. If exploration of the anus gives negative results, (neither external hæmorrhoids nor fissure) and there are general symptoms pointing to rectal lesion, the diagnosis is between internal hæmorrhoids and polypus beyond recognition by ordinary exploratory procedures. Hence other means of recognition must be adopted.

An important sign of fissure is the unusual resistance encountered by the finger in overcoming the sphincter. When the finger has been previously well oiled, and is introduced as gently as possible, and yet the infant cries with pain, the existence of fissure may almost be affirmed, and the characteristic lesion is almost always encountered.

5. *Successful Capillary Puncture of the Liver — Urticaria.* LAVERAN. (*Lyon Médic.*, No. 21.)

L. reported to the Medical Society of the Paris Hospitals a case of successful puncture of the liver, followed by urticaria. Lereboullet assigns the urticarial symptoms to penetration of the contents of the invaded cysts into the peretoneal cavity. But Labbé had a patient with hydatia cyst of the liver, who suffered from urticaria *before* puncture was tried. Urticaria is frequent in all disorders of the gastro-hepatic apparatus in icterus by retention, and in any condition of irritation of the splanchnic nerves. Tcauzoni has noticed its appearance after the application of leeches to the neck of the uterus in hysterical patients. Leopold, after leeching over the sacrum. Damaschino has seen urticaria in the cases where puncture had not completely emptied the cyst.

